# Chronic urticaria and thyroid autoimmunity: a perplexing association

**DOI:** 10.1093/omcr/omx099

**Published:** 2018-02-22

**Authors:** Subothini Sara Selvendran, Nikhil Aggarwal

**Affiliations:** Imperial College London, London SW7 2AZ, UK

## Abstract

Chronic urticaria has long been thought to be associated with autoimmune conditions, in particular autoimmune thyroid disease (AITD). We detail an unusual case of a 49-year-old patient presenting with urticaria distributed on both shins and hands, with no known associated triggers, and subsequently diagnosed with AITD. The urticaria resolved upon treatment of the AITD. We also summarize the currently postulated pathophysiological links between the two diseases. This case highlights that physicians should have a low threshold for investigating autoimmune conditions in cases of chronic urticaria, with particular attention given to AITD.

## INTRODUCTION

The term urticaria is commonly used to describe a skin condition characterized by recurrent attacks of itchy hives that may vary in size, number and distribution [1]. Urticaria is defined as chronic when patients experience daily manifestations for more than 6 weeks [2]. In total, 40–50% of these patients have accompanied angioedema [2]. There are several known causes of chronic urticaria (CU); some of the most important include contact, allergy and stress [2]. Autoimmunity due to IgE and IgG autoantibodies are also thought to play a significant role in the aetiology of CU in a sub-population of patients [1]. In particular, the association of thyroid autoimmunity with CU has been described since 1907 though the extent of association and mechanistic links are not fully understood [1]. We report a patient presenting with persistent hives with no associated triggers, and eventually diagnosed with autoimmune thyroid disease (AITD). Additionally, we explore and summarize the postulated pathophysiological links between CU and thyroid autoimmunity.

## CASE REPORT

**Figure 1:**
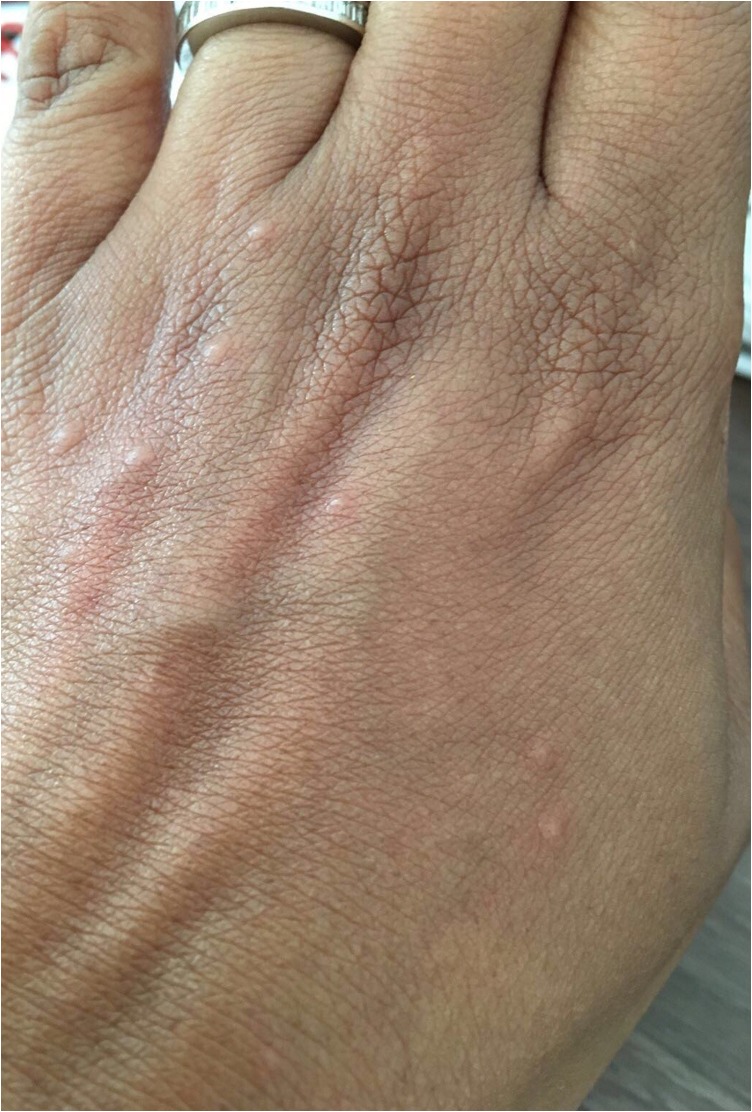
Urticaria after 1 week of treatment.

A 49-year-old woman presented with a 6-week history of pruritic raised wheals on her shins and dorsum of both hands. The lesions appeared suddenly and lasted from between 8 and 12 h, with no association to the time of day. The size varied from between 3 and 15 mm in diameter. The patient was unaware of any triggering factors such as specific foods, cold or hot temperatures, water or emotional stress. However, the patient reported increased lethargy. Examination revealed urticarial wheals but was otherwise completely unremarkable with vital signs within normal limits (Fig. 1).

**Figure 2:**
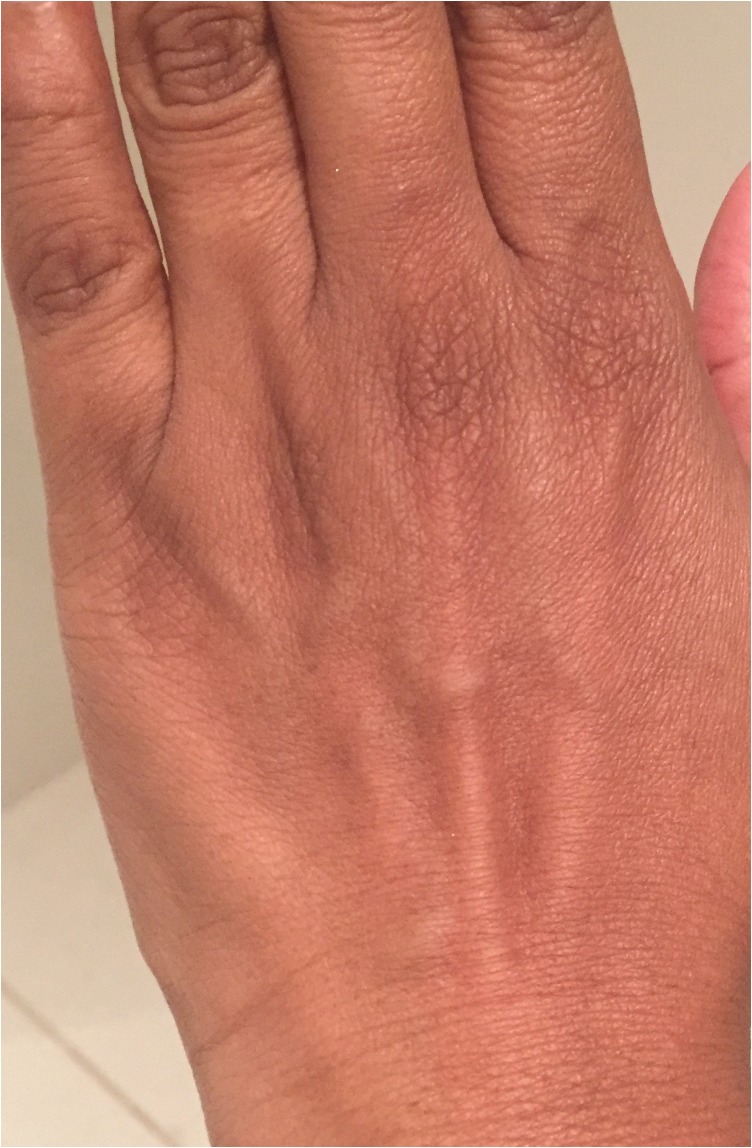
Absence of urticaria after 4 weeks of treatment.

Due to the patient’s associated symptom of lethargy and in accordance with the British Society for Allergy and Clinical Immunology guidelines [3], a complete blood count, erythrocyte sedimentation rate (ESR), liver function tests (LFTs) and thyroid function tests were measured to rule out the possibility of any systemic disease. Investigations revealed normal haemoglobin, white cell count, absence of eosonophillia, normal ESR and normal LFTs. However, the T4 and T3 was lower than the normal range (8.6 and 3.2 pmol/L, respectively [normal T4 values: 10–20pmol/L and normal T3 values: 3.5–7.8 pmol/L]) and the thyroid stimulating hormone (TSH) was raised (5.4 mU/L [normal range: 0.4–4 mU/L]). A diagnosis of hypothyroidism was made. Given the association described in the literature and to help elucidate the possible trigger of the urticaria, a further investigation was conducted which revealed raised anti-thyroid peroxidase antibodies (48 IU/mL [normal values: <38 IU/mL]) confirming AITD. Treatment was commenced with 25 µg once daily thyroxine replacement and Cetirizine 10 mg once daily for 4 weeks was started to manage the urticaria. The patient was reviewed 4 weeks later. By this time the urticaria had resolved and the patient was managed on long-term thyroxine replacement. There have been no relapses of urticaria after 14 months of successful management of AITD, further demonstrating this association (Fig. 2).

## DISCUSSION

Approximately 5–34% of patients with CU have anti-thyroid antibodies and another 5–10% have clinically or biochemically apparent thyroid disease [4]. The connection between AITD and CU is an unresolved mystery but there are a number of plausible explanations.

CU is initiated by inappropriate activation and degranulation of mast cells, a key pathophysiological event [5]. A possible mechanistic link between CU and thyroid autoimmunity is thought to involve elevated IgG anti-thyroid autoantibodies (IgG-anti-TPO, IgG-anti-TG) [4]. It has been postulated that IgG anti-thyroid autoantibodies are not directly involved in the degranulation of mast cells but may intensify mast cell susceptibility to other activating signals [4]. Rumbyrt *et al.* [6] theorized that an inflammatory response in the thyroid gland leads to a generalized inflammatory state and decreases the activation threshold of mast cells to other stimuli. Products of the thyroid autoimmune response such as thyroid protein immune complexes stimulate the classical complement pathway, leading to the C3a and C5a generation. This activates mast cells and basophils in CU patients [4].

Recently, studies have demonstrated high levels of IgE anti-thyroid autoantibodies (IgE anti-TPO and IgE anti-dsDNA) in some CU patients [2]. It is thought that IgE anti-TPO autoantibodies, when bound to the surface of mast cells and basophils, directly increase their sensitivity to specific circulating antigens found in autoimmune thyroid damage such as thyroid peroxidase [2]. This is further reinforced by the efficacy of omalizumab, an anti IgE therapy, in CU patients who are IgE anti-TPO-positive [7].

Infectious agents are thought to be involved in the pathogenesis of both CU and thyroid autoimmunity [4]. For example, *Staphylococcus aureus* protein A has shown to cause the release of pro-inflammatory cytokines that induce attacks of CU [4]. Moreover, Wan *et al.* [8] illustrated that staphylococcal enterotoxin A superantigen stimulation of thyroglobulin primed cells caused the adoptive transfer of experimental autoimmune thyroiditis in mice. In addition to this, Hepatitis C virus has been proposed to play a role in the aetiology and pathogenesis of urticaria and AITD [4]. Marone *et al.* [9] demonstrated that protein Fv, produced by viral hepatitis, stimulates urticaria reactions by binding to a component of IgE. Additionally, Hepatitis C envelope glycoproteins, E1 and E2, are thought to activate intracellular signalling pathways triggering the release of pro-inflammatory mediators such as interleukin-8. This could possibly induce thyroid autoimmunity through bystander activation [10].

Finally, recent studies have highlighted the overlap of immunological mechanisms that play a role in the pathogenesis of both CU and thyroid autoimmunity [11]. Interleukin-6 (IL-6) has been linked with the development or exacerbation of CU [11]. It has also been discovered in high levels in trials studying AITD. Interestingly, a correlation between IL-6 and severity of CU as well as anti-TPO levels has been demonstrated [11]. Additionally, lower serum levels and functionality of T-CD4+CD25+Foxp3+ cells are associated with the pathogenesis of these two diseases [11]. Further studies are needed to confirm these hypothetical observations.

In conclusion, there is a wide array of potential processes by which the two conditions are interlinked. Therefore, physicians and other healthcare workers should have a low threshold for investigating autoimmune conditions, in particular AITD, with a patient presenting with CU.
